# Potential Future Use, Costs, and Value of Poliovirus Vaccines

**DOI:** 10.1111/risa.13557

**Published:** 2020-07-09

**Authors:** Kimberly M. Thompson, Dominika A. Kalkowska

**Affiliations:** ^1^ Kid Risk, Inc. Orlando FL USA

**Keywords:** financial risk, polio eradication, vaccine

## Abstract

Countries face different poliovirus risks, which imply different benefits associated with continued and future use of oral poliovirus vaccine (OPV) and/or inactivated poliovirus vaccine (IPV). With the Global Polio Eradication Initiative (GPEI) continuing to extend its timeline for ending the transmission of all wild polioviruses and to introduce new poliovirus vaccines, the polio vaccine supply chain continues to expand in complexity. The increased complexity leads to significant uncertainty about supply and costs. Notably, the strategy of phased OPV cessation of all three serotypes to stop all future incidence of poliomyelitis depends on successfully stopping the transmission of all wild polioviruses. Countries also face challenges associated with responding to any outbreaks that occur after OPV cessation, because stopping transmission of such outbreaks requires reintroducing the use of the stopped OPV in most countries. National immunization program leaders will likely consider differences in their risks and willingness‐to‐pay for risk reduction as they evaluate their investments in current and future polio vaccination. Information about the costs and benefits of future poliovirus vaccines, and discussion of the complex situation that currently exists, should prove useful to national, regional, and global decisionmakers and support health economic modeling. Delays in achieving polio eradication combined with increasing costs of poliovirus vaccines continue to increase financial risks for the GPEI.

## 1 INTRODUCTION

Not long after the introduction of oral poliovirus vaccine (OPV) in the 1960s, national immunization strategies in countries that achieved high coverage in all subpopulations experienced significant reductions in the transmission of wild polioviruses (WPVs). Countries and the region of the Americas achieved complete die out of WPVs, which led to significant estimated health and financial benefits (Musgrove, 1988; Thompson & Duintjer Tebbens, 2006). These successes motivated the 1988 World Health Assembly to resolve to eradicate WPVs and end all cases of poliomyelitis (World Health Assembly, 1988), and to the launch of the Global Polio Eradication Initiative (GPEI). At the time, countries expected to stop all poliovirus vaccination after ending the transmission of WPVs to realize an eradication “dividend,” as occurred with smallpox (Barrett, 2004). Now, as the GPEI enters its fourth decade and faces criticisms about delays and high cumulative costs, countries continue to face difficult choices about their national polio immunization strategies in the increasingly complicated polio endgame.

Prior to the Coronavirus Disease 2019 (COVID‐19) pandemic, the risk of insufficient support for the GPEI (i.e., financial risks) posed a threat to the GPEI and polio eradication. Despite full support from donors of its 2013–2018 Strategic Plan (World Health Organization Global Polio Eradication Initiative, 2013), the GPEI did not deliver on achieving polio eradication by 2020 (Thompson & Kalkowska, 2020). The GPEI released a 2019–2023 Strategic Plan with a $4.2 billion budget (World Health Organization Global Polio Eradication Initiative, 2019), but financial risks remain a concern, and the COVID‐19 pandemic will likely further delay eradication and increase costs. Given the financial risks, understanding the future costs of poliovirus vaccines and the economic value of the polio cases that they prevent require the use of updated cost and valuation inputs.

## 2 POLIO VACCINES

Unlike many other vaccine‐preventable diseases, polio vaccination can occur with either or both of two vaccines with different benefits, risks, and costs: OPV and inactivated poliovirus vaccine (IPV) (Duintjer Tebbens & Thompson, 2017b). The relatively cheap‐to‐produce and deliver live, attenuated OPV infects recipients who can secondarily infect and induce or boost immunity in others in the community. OPV infection induces mucosal intestinal immunity, provides life‐long protection from paralysis (if reinfected), and reduces the probability, duration, and infectiousness of reinfection. Unfortunately, the risks of using OPV include a small, but nonzero probability of vaccine‐associated paralytic polio (VAPP) in a small fraction of individuals first exposed to poliovirus by OPV (on the order of 1 in a million) (Duintjer Tebbens, Pallansch, et al., 2006; Platt, Estivariz, & Sutter, 2014). In addition, a small number of individuals with some B‐cell‐related primary immunodeficiencies develop infections with immunodeficiency‐associated vaccine‐derived polioviruses (iVDPVs) and they may excrete live polioviruses for prolonged periods of time (Duintjer Tebbens, Pallansch, et al., 2006; Duintjer Tebbens, Pallansch, & Thompson, 2015; Kalkowska, Pallansch, & Thompson, 2019; Kew et al., 1998). Finally, OPV transmission in populations with low immunity can lead to the loss of the attenuating mutations of OPV, and with continued transmission, these OPV‐related viruses may evolve to become circulating VDPVs (cVDPVs) that can cause outbreaks and behave like WPVs (Duintjer Tebbens, Pallansch, et al., 2006; Duintjer Tebbens, Pallansch, Kim, et al., 2013; Kew et al., 2002). Compared to OPV, injectable killed IPV costs significantly more to produce and deliver. IPV production involves growing large quantities of WPVs (Salk IPV) or OPV (Sabin IPV) followed by inactivation. IPV protects only the vaccine recipient from paralysis and provides mainly humoral immunity, which does not significantly reduce the probability, duration, or infectiousness of an intestinal infection (Duintjer Tebbens et al., 2013a, 2013b; Thompson & Duintjer Tebbens, 2014).

Although both OPV and IPV offer life‐long protection to vaccine recipients from paralysis, they differ significantly in their ability to stop and prevent live poliovirus transmission in populations. Following the early introduction of OPV in the 1960s and through 2004, countries that included OPV in their national immunization programs exclusively used trivalent OPV (tOPV), which contains all three serotypes, for all routine immunization (RI) and supplemental immunization activities (SIAs). IPV only comes in a trivalent formulation (i.e., with all three serotypes).

While WPVs circulated broadly (causing paralysis in approximately 1/200, 1/2,000, and 1/1,000 first infections with WPV serotypes 1, 2, and 3, respectively (Duintjer Tebbens, Pallansch, Wassalik, Cochi, & Thompson, 2015; Nathanson & Kew, 2010), the VAPP risks of OPV paled in comparison to WPV risks. However, as countries eliminated WPVs, OPV risks become more visible. Notably, in high‐income countries, concerns about the small, but nonzero risks of VAPP led to a switch from using OPV to the use of a sequential IPV/OPV schedule (which protects vaccine recipients from VAPP by making the vaccine recipient's first exposure IPV, but still leads to the induction of intestinal mucosal immunity from subsequent OPV doses) or an IPV‐only schedule, albeit at a high cost (Miller, Sutter, Strebel, & Hadler, 1996; Thompson & Duintjer Tebbens, 2006). These countries still faced concerns about importation of live polioviruses due to ongoing global transmission, and they could not safely stop immunization. Following the trend started by the U.S. shift from using OPV only to a sequential IPV/OPV RI schedule to prevent VAPP and then to an IPV‐only RI schedule, other high‐ and upper middle‐income countries increasingly began using IPV (Thompson et al., 2013). However, Israel, which moved to an IPV‐only schedule in 2005, reintroduced OPV into an IPV/OPV sequential schedule in 2014. This national restart of bOPV use followed the 2013 detection of WPV1 transmission and the inability to stop this transmission using IPV only (Kalkowska et al., 2015). This incident provided an important lesson for why continued OPV use may still be important in some relatively higher income countries.

Despite the relatively small, but nonzero risks of VAPP, countries that eliminated WPV nationally and continue to use OPV must continue to maintain high coverage. The use of OPV with low coverage leaves the unvaccinated individuals and the population at risk of paralysis from outbreaks of reestablished transmission of an imported WPV and/or development and outbreaks of cVDPVs.

## 3 EVOLUTION OF GLOBAL POLIOVIRUS VACCINE RECOMMENDATIONS

National health leaders make the decisions for their countries, which leads to a wide range of polio immunization schedules for RI and some use of SIAs in some countries (Thompson et al., 2013). However, the World Health Organization (WHO) Strategic Advisory Group of Experts (SAGE) on Immunization makes some recommendations, and countries that depend on external financing also consider relevant donor recommendations, preferences, and/or requirements (e.g., those who submit funding applications to Gavi, the Vaccine Alliance (Geneva, Switzerland).

With the last serotype 2 WPV (WPV2) case reported in 1999, in the mid‐2000s, the GPEI encouraged vaccine manufacturers to relicense monovalent OPV (mOPV) for serotypes 1 and 3. Starting in 2005, the GPEI and some endemic countries decided to preferentially use serotype 1 mOPV (mOPV1), and then later also serotype 3 mOPV (mOPV3) in some SIAs. This usage assumed that individual children vaccinated in these SIAs would more likely “take” to serotype 1 (or 3) than if tOPV were used, because of interference by the serotype 2 OPV in tOPV, and that using mOPVs would accelerate ending transmission of WPVs (Grassly et al., 2007). However, this strategy ignored the population impacts. After the use of mOPV1 opened up immunity gaps that supported transmission and outbreaks of serotype 3 WPV (WPV3) and necessitated the use of mOPV3, the GPEI supported the development of bivalent OPV (bOPV, containing serotypes 1 and 3). Starting in 2010, some countries that had been using mOPVs for some SIAs started using bOPV for some SIAs instead. Importantly, however, these countries still used tOPV for RI everywhere and for most SIAs, while other countries continued exclusive use of tOPV. The immunity gaps created by the mOPV and bOPV use for some SIAs led to a dramatic shift of observed cVDPVs from a larger number of serotype 1 cVDPV (cVDPV1) cases prior to the mid‐2000s (Duintjer Tebbens, Pallansch, et al., 2006) to many more serotype 2 cVDPV (cVDPV2) cases after the introduction of mOPV1 and mOPV3 use (Duintjer Tebbens, Pallansch, Kim, et al., 2013). Moreover, the preferential mOPV and bOPV use in SIAs in endemic countries did not accelerate the disruption of WPV1 or WPV3 transmission (Kalkowska, Duintjer Tebbens, & Thompson, 2014a, 2014b; Thompson & Duintjer Tebbens, 2015).

Appreciation of the risks of using OPV after successful WPV eradication motivated the strategy of globally coordinated OPV cessation after the certification of WPV eradication (World Health Assembly, 2008). Given delays in WPV1 eradication, the GPEI decided to first globally phase out serotype 2 OPV (OPV2) in late April–May 2016 (World Health Organization, 2016a, 2016b). Adoption of this OPV cessation strategy assumed that prior to OPV cessation, countries would achieve and maintain high levels of population immunity to transmission, which would minimize the risks of failure of the strategy and the need to restart any serotype of OPV after its coordinated global cessation (Duintjer Tebbens, Pallansch, Wassalik, et al., 2015).

An early discussion of the vaccine choices post WPV‐eradication identified the different sets of decisions for countries with different starting points (Sangrujee, Duintjer Tebbens, Cáceres, & Thompson, 2003). A 2008 economic analysis of the polio endgame recognized the potential role of IPV for the polio endgame, but emphasized that its high cost and relatively small expected health benefits after WPV eradication made IPV not cost‐effective (Thompson et al., 2008). A 2012 study about national polio immunization strategies discussed IPV use as a potential option after OPV cessation, but *not as a prerequisite* to OPV cessation, and again noted the need for a low‐cost IPV for relatively lower income countries to make IPV a reasonable option (Thompson & Duintjer Tebbens, 2012). In 2014, the GPEI and SAGE recommended that all national immunization programs introduce 1 dose of IPV into their RI schedules at least six months prior to OPV2 cessation (i.e., by the end of 2015) and *made IPV introduction a prerequisite* to OPV2 cessation (World Health Organization, 2014a). The recommendation came without any supporting economic analyses, and anticipated sufficient IPV supply at a relatively low cost. However, the introduction of IPV into OPV‐using countries proved much more challenging than expected. Scale‐up of production by the existing producers came with delays (i.e., it proved more challenging than anticipated), and instead of a flood of new manufacturers making a significantly lower‐cost IPV, IPV supply remained constrained and prices stayed higher than hoped over the past several years, followed by an IPV price increase in 2019. The actual experience demonstrated the unrealistic and optimistic assumptions made by the GPEI about anticipated IPV supply and cost, which provided an important lesson.

To deal with IPV shortages between 2015 and 2019, the GPEI encouraged and some countries explored fractional IPV (i.e., delivery of one‐fifth of a full dose), which these countries delivered intradermally. Remarkably, however, despite increasing IPV prices and insufficient supply for one dose of IPV globally, in 2017, the GPEI and SAGE recommended that all countries use at least two IPV doses for at least 10 years after cessation of the last OPV serotype and again suggested this as a prerequisite to the ultimate cessation of all OPV use in RI (World Health Organization, 2017). Notably, this recommendation also came without any economic or risk analyses to support it, and without consideration of the opportunity costs of national immunization programs investing in insurance for long‐term protection from paralysis from an eradicated disease instead of other potential investments (i.e., more doses of other vaccines or greater investment in other health interventions). The recommendation primarily focused on the concept that two doses of IPV would provide better (i.e., potentially more durable) protection from paralysis than one dose of IPV in the long term. Thus, without any discussion about the financial consequences, the GPEI and SAGE implicitly changed their global baseline to a strategy of high control with two doses of IPV (independent of eradication). Notably, a recent cost analysis assumed very high control with 2 doses of IPV in perpetuity (Zimmermann, Hagedorn, & Lyons, 2020) as the reference case (or *status quo*), in contrast to control with OPV that prior economic analyses assumed (Thompson & Duintjer Tebbens, 2007; Duintjer Tebbens, Pallansch, Wassalik, et al., 2015; Thompson et al., 2008) and which still represents a possible option. Finally, the GPEI encouraged and some countries used IPV in some SIAs, despite modeling results that demonstrated this as not effective for stopping or preventing transmission and not cost‐effective (Duintjer Tebbens & Thompson, 2017a). During a time of IPV shortages as supply ramped up between 2016 and 2018, this IPV use in outbreak response SIAs competed with giving IPV doses to children in other countries in RI programs that achieve higher coverage.

## 4 UNCERTAINTIES RELATED TO POLIOVIRUS VACCINE DEMAND

In the current polio endgame, poliovirus vaccine manufacturers face considerable uncertainty about future demand from some segments of the market. While high‐ and upper middle‐income countries will likely continue IPV use for the foreseeable future, relatively lower income countries that prefer a much cheaper and easier‐to‐deliver vaccine may prefer to continue to use only OPV, particularly if they do not receive external financing. In addition, those countries that use IPV may prefer to deliver fractional doses, which use substantially less antigen and implies significant savings on vaccine costs for the countries, but leads to less demand for IPV manufacturers. Not surprisingly, IPV manufacturers would most likely much prefer the certainty associated with a SAGE recommendation and commitments for external financing for IPV formulated in a combination vaccine that provide some guarantee of a sustainable market (Thompson & Duintjer Tebbens, 2014). A combination IPV formulation uses the most IPV antigen (e.g., four doses in the RI schedule and all full IPV doses), and thus creates the highest global demand for IPV‐containing products that would come with price premiums. High‐income countries increasingly prefer combination vaccines that include more antigens because these formulations reduce the total number of needle sticks required, which saves on vaccine administration costs and reduces events associated with injections. However, the production of combination vaccines containing IPV remains challenging, and becomes complicated by the preferred formulation and timing of delivery of some other components in the combination product (e.g., acellular vs. whole cell pertussis, early infant protection vs. reduced efficacy associated with maternal antibodies). In addition, early work on IPV in a vaccine patch presentation shows promise for both fractional dosage and ease of delivery (Anand et al., 2015; Edens, Dybdahl‐Sissoko, Weldon, Oberste, & Prausnitz, 2015; Muller, Fernando, et al., 2017; Muller, Pearson, et al., 2016). Thus, although the SAGE recommendation would seem to imply a large, long‐term market for IPV, the nature of that market remains highly uncertain as the eradication of all WPVs continues to take more time, the GPEI costs continue to increase, and countries fail to stop cVDPV2s after OPV2 cessation increasing the chances of needing to restart OPV2, potentially in a tOPV formulation (Duintjer Tebbens & Thompson, 2018; Thompson & Kalkowska, 2019; Kalkowska et al., 2020b).

In addition to uncertainty about IPV costs and supply, with the delay of eradication, significant uncertainty now exists about the future of OPV demand, and thus, OPV costs and supplies. Some OPV manufacturers already stopped their OPV bulk production consistent with the GPEI expectation of ending all WPV1 transmission in 2019 (World Health Organization Global Polio Eradication Initiative, 2013) (and prior missed GPEI WPV eradication deadlines). However, global reporting of WPV1 cases in 2019 exceeded the number of cases in 2018 (World Health Organization, 2020), and recent modeling suggests that the GPEI is not on track to eradicate WPV1 by 2024 with its current plans (Kalkowska, Wassilak, Cochi, Pallansch, & Thompson, 2020a). As the incidence of cVDPV2 cases continues to increase, the possibility and complexity of needing to restart OPV2 bulk production also appears more likely (Kalkowska et al., 2020a, 2020b; Thompson & Kalkowska, 2019), in addition to maintaining the use of bOPV until the time of bOPV cessation. Moreover, the Global Certification Commission recently certified the eradication of WPV3 (World Health Organization, 2019a), which creates the possibility of globally coordinated serotype 3 OPV (OPV3) cessation prior to serotype 1 OPV (OPV1) cessation. Adding further complexity, accelerated efforts to develop a novel OPV strain for serotype 2 (nOPV2), which developers genetically engineered to make less likely to revert to the neurovirulent genotype than Sabin OPV2, may lead to yet another option (Van Damme et al., 2019). With only one current WHO prequalified OPV manufacturer still willing and able to make OPV2 bulk, new OPV2 bulk production competing with the bulk production for OPV1 and OPV3, and all OPV bulk competing for the same OPV filling lines, the logistics of managing the global OPV supply remain a challenge. In addition, the failure of Pakistan and Afghanistan to order bOPV supplies planned and produced for them in 2019 (i.e., due to their decisions not to conduct some bOPV SIAs) further exacerbates the OPV supply situation, and contributes to continued failure to stop WPV1 transmission there (Kalkowska & Thompson, 2020).

The current complexity of the polio endgame leads to many questions related to poliovirus vaccines, including: Which, if any, polio vaccines will countries use prior to and after WPV eradication, and for how long after eradication? Will eradication of all three serotypes of WPVs occur? Will the GPEI partners remain committed to financially supporting polio eradication until success? How long will OPV be needed to stop outbreaks? Will the world need to restart the production of OPV2? Will this lead to changes in the strategy of stopping all OPV use after WPV eradication? What new poliovirus vaccines might lead to changes in preferences? How will countries finance current and future poliovirus vaccines in their national immunization programs? How much will future poliovirus vaccines cost? The remainder of this analysis explores the historical and potential future cost and valuation inputs to support modeling efforts that may address some of these questions.

In April 2019, the GPEI issued a new strategic plan for 2019–2023, with a $4.2 billion budget (World Health Organization Global Polio Eradication Initiative, 2019). However, recognizing that this plan did not address the continued serotype 2 transmission occurring (now four years after globally coordinated OPV2 cessation), the GPEI also issued a new cVDPV2 strategy (World Health Organization Global Polio Eradication Initiative, 2020).

## 5 POLIO VACCINE COST AND VALUATION INPUT ESTIMATES

**Fig 1:**
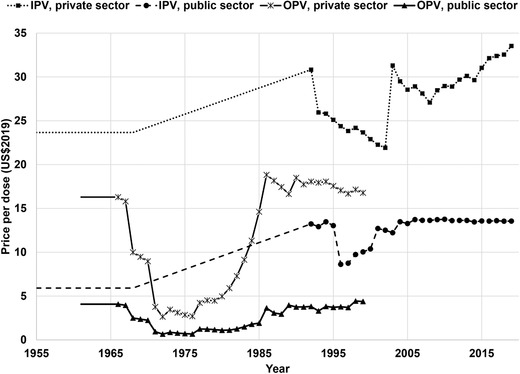
Price per dose of poliovirus vaccine in the US in real 2019 dollars (US$2019) by vaccine type* and sector (Bureau of Labor Statistics, 2020; Centers for Disease Control and Prevention, 2019; Thompson & Duintjer Tebbens, 2006). **Note**: * IPV in this figure includes the original IPV vaccine developed by Salk and enhanced potency IPV (eIPV) as a standalone vaccine currently used (see text about context related to IPV‐containing combination vaccines).

Numerous prior economic analyses related to polio vaccines made assumptions about polio vaccine costs. For this analysis, to ensure consistent comparisons, we convert all financial estimates to 2019 US dollars (US$2019) by using the US Consumer Price Index (CPI) (Bureau of Labor Statistics, 2020). Costs vary by country, vaccine formulation, and time, and sometimes within the public and private sector within a country. For example, Fig. 1 shows the time trends for US polio vaccine cost estimates for the public and private sectors prior to 2001 (Thompson & Duintjer Tebbens, 2006) and from 2001 on (Centers for Disease Control and Prevention, 2019) inflated using the CPI (Bureau of Labor Statistics, 2020). As shown in Fig. 1, costs may differ significantly for vaccines and sectors. When use of a vaccine ends, as occurred with OPV in the US in 1999, this corresponds to no prices at all. In the US, the addition of IPV‐containing combination vaccines leads to more complexity than captured in Fig. 1. Fig. 1 reports the price of eIPV (i.e., IPOL®) for the IPV price trends, and not the weighted price of all IPV vaccines used, which we could not obtain. Comparison of the incremental cost of adding IPV to a combination vaccine in 2008 (i.e., Pentacel®, which contains DTaP‐Hib‐IPV, compared to TriHIBit®, which contains DTaP‐Hib) implied an IPV antigen component cost of approximately $23.22 (public) and $30.10 (private) in the more complicated combination vaccine (Centers for Disease Control and Prevention, 2008). This price for the IPV component in the combination vaccine exceeded the standalone IPV price (i.e., IPOL®) by approximately $10 per dose (i.e., 100% higher price for the public sector and approximately 30% higher price for the private sector). The costs of administering vaccine also differ for the public and private sectors (Zhou et al., 2014).

For purposes of global economic analyses, we ignore differences in vaccine costs within a country. We stratify countries according to their World Bank Income Level (WBIL): high‐, upper middle‐, lower middle‐, and low‐income countries (i.e., HI, UMI, LMI, and LI countries, respectively) (World Bank, 2019). For prospective modeling, we assume that countries will remain in the 2018 WBIL (World Bank, 2019). We note that our prior similar prospective assumptions that used 2002 WBIL classifications prospectively (Duintjer Tebbens et al., 2011) did not capture substantial changes in large countries (e.g., India shifting from LI to LMI in 2009 and China and the Russian Federation shifting from LMI to UMI in 2012 and 2006, respectively). However, offsetting the shifts of these countries to higher income categories (i.e., leaving them in relatively lower WBILs, effectively kept their vaccine prices lower, which was consistent with these countries self‐producing their own poliovirus vaccines). Overall, although this strategy ignores multiple sources of variability, we assume that the differences between income levels represent important strata. Thus, this approach captures some high‐level variability in conditions throughout the world with respect to economic benefits and costs.

We caution that we cannot know in advance what manufacturers will offer or health ministers in individual countries will see as actual future options with market prices. In addition, as with all products, prices will depend on the scale of production, with large markets supporting lower prices per dose due to economies of scale. However, to support global modeling of options, we need to develop updated projections that reflect the current situation, and we anticipate that our transparency in developing the estimates will also prove useful to other polio vaccine stakeholders. Table I summarizes estimates of polio vaccine cost and valuation inputs for different income levels based on prior analyses and useful for retrospective economic analyses for 1988–2018 (Duintjer Tebbens & Thompson, 2016; Duintjer Tebbens et al., 2011; Duintjer Tebbens, Pallansch, Wassalik, et al., 2015; Duintjer Tebbens, Sangrujee, & Thompson, 2006; Thompson & Duintjer Tebbens, 2014; Thompson et al., 2008) after CPI adjustment (Bureau of Labor Statistics, 2020) to US$2019. As shown in Table I, we use different vaccine administration costs for the different vaccine formulations (OPV vs. IPV), because IPV administration requires trained health personnel, while volunteers can deliver OPV.

**Table I:** Economic Model Inputs for Retrospective Global Modeling Based on Our Prior Estimates Stratified by World Bank Income Level Inflated to 2019 United States Dollars ($) Only (No Other Adjustments) (Duintjer Tebbens & Thompson, 2016; Duintjer Tebbens et al., 2011; Duintjer Tebbens, Pallansch, et al., 2006; Duintjer Tebbens, Pallansch, Wassalik, et al., 2015; Thompson & Duintjer Tebbens, 2014; Thompson et al., 2008)

Input	LI	LMI	UMI	HI
Vaccine price per dose				
‐ OPV (any formulation), multidose vial	$ 0.129	$ 0.129	$ 0.142	$ 7.107
‐ IPV, full dose, standalone, multidose vial	$ 1.421	$ 2.487	$ 3.553	$ 14.21
‐ IPV, full dose, combination or single dose	$ 2.744	$ 4.939	$ 6.585	$ 27.11
Administration costs per dose				
‐ OPV in RI	$ 0.95	$ 0.95	$ 2.51	$ 3.18
‐ IPV given with OPV in RI (incremental)	$ 1.00	$ 1.00	3.00	NA
‐ IPV single antigen in RI (full or fractional)	$ 1.78	$ 1.78	$ 4.69	$ 17.06
‐ IPV combination in RI	NA	NA	$ 1.17	$ 4.26
‐ OPV in pSIAs	$ 0.66	$ 0.66	$ 3.62	$ 4.61
‐ OPV in oSIAs	$ 0.99	$ 0.99	$ 5.43	$ 6.91
‐ IPV in SIAs	$ 1.78	$ 1.78	$ 4.69	$ 17.05
Number of doses in RI schedule				
‐ OPV‐only (pre‐2015)	3	3	3	NA
‐ OPV+IPV (started in 2015)	3 +1	3 +1	3 +1	NA
‐ IPV/OPV sequential	2 + 2	2 + 2	2 + 2	3 + 1
‐ IPV‐only standalone (post OPV use in RI)	1 or 2	1 or 2	1 or 2	3
‐ IPV‐only, combination	3	3	3	3
Effective vaccine wastage				
‐ OPV in RI	20%	20%	15%	10%
‐ IPV (10‐dose vial) in RI	15%	15%	10%	5%
‐ IPV (10‐dose vial) in IPV/OPV RI	30%	30%	20%	10%
‐ OPV or IPV in SIAs	15%	15%	10%	10%
Treatment costs per case	$ 711	$ 7,110	$ 71,100	$ 711,000

HI = high‐income; IPV = inactivated poliovirus vaccine; LI = low income; LMI = lower middle income; N/A = not applicable; OPV = oral poliovirus vaccine; oSIA = outbreak response SIA; pSIA = planned, preventive SIA; RI = routine immunization; SIA = supplemental immunization activity; UMI = upper middle income; WBIL = World Bank Income Level.

The historical cost estimates for poliovirus vaccines in Table I do not account for some of the products that have entered or may enter the market. In addition, for prospective health economic analyses, we also recognize the opportunity to make adjustments based on updated price estimates from the UNICEF supply division (UNICEF, 2019), policies that affect wastage assumptions (World Health Organization, 2014b), information about the costs of new products (Leo, 2019; Mvundura et al., 2019), and the need to consider the implications of updated guidelines and recommendations (World Health Organization, 2019b).

Table II summarizes our best estimates of polio vaccine cost and valuation inputs for different income levels considering the information available on January 1, 2020. Our estimates for vaccine antigen price per dose in Table II consider actual price lists (UNICEF, 2019) and discussions with manufacturers, and thus, they differ from demand‐side forecasting prices used by other analysts (e.g., Gavi price forecasts [Portnoy et al., 2015; Zimmermann et al., 2020]), which have tended to fall below actual prices for IPV. As shown in Table II, our estimates suggest higher prices for the OPV formulations than the estimates in Table I. This reflects consolidation of the market and increased containment requirements. In addition, in the absence of an existing nOPV product, we assume a price for nOPV of twice the price of existing, licensed OPV, although we recognize that the future price could be higher or as low as current OPV. Although our global modeling assumes that no high‐income countries use OPV in RI (Kalkowska et al., 2020a), we included an OPV price for HI in Table II for purposes of comparison because its use remains theoretically possible. In Table II, we include an estimate for the IPV price as a stand‐alone vaccine, or as part of a combination vaccine, for which we assume a hexavalent formulation. Although we previously assumed no price premium for the inclusion of the IPV component in a combination formulation, Table II shows a higher cost for the IPV component in a combination vaccine compared to IPV in a stand‐alone formulation. Given uncertainty about the prospects of using complex combination vaccines for LI and LMI countries, we assume a relatively higher price for the IPV component in hexavalent formulations than as a standalone formulation, consistent with announcements made by vaccine manufacturers related to serving developing country markets (Leo, 2019). We anticipate the availability of hexavalent IPV‐containing vaccines for LI and LMI in 2023, although some doses may become available early or it may take longer to scale‐up production. Finally, we include a price for IPV stand‐alone formulated as a vaccine patch, which we assume would take advantage of dose sparing, and could become available as early as eight years after the initiation of concerted efforts to develop it (e.g., as early as 2029 if development starts in 2021). For HI countries, we assume that an IPV vaccine patch would cost the same for the vaccine per dose as the IPV component of a hexavalent combination vaccine.

**Table II:** Prospective Economic Model Inputs (for 2019 on) by World Bank Income Level for Vaccine, Treatment, and Societal Costs in 2019 United States Dollars ($) Based on Evidence and Guidance Available as of January 1, 2020

Input	LI	LMI	UMI	HI
Number of countries	31	48	54	68
Number of people (millions)	724	3,065	2,709	1,215
Number of children under five years old (millions)	112	313	188	65
Number of surviving infants (millions)	24	63	37	13
Vaccine price per dose				
‐ OPV (any formulation)	$ 0.15	$ 0.15	$ 0.33	$ 8.75*
‐ nOPV (formulations containing any nOPV)	$ 0.30	$ 0.30	$ 0.66	$ 8.75*
‐ IPV, full dose, standalone	$ 2.50	$ 2.65	$ 4.75	$ 14.27
‐ IPV, fractional dose, standalone	$ 0.50	$ 0.53	$ 0.95	NA
‐ IPV component, combination, full dose	$ 3.50	$ 4.00	$ 6.58	$ 27.11
‐ IPV, vaccine patch (dose‐sparing)	$ 1.70	$ 1.73	$ 2.95	$ 27.11
Administration costs per dose				
‐ OPV in RI or SIAs	$ 0.95	$ 0.95	$ 2.51	$ 3.18*
‐ IPV given with third OPV dose in RI (full)	$ 1.00	$ 1.00	$ 3.00	NA
‐ IPV single antigen in RI or SIAs	$ 1.78	$ 1.78	$ 4.69	$ 17.06
‐ IPV combination (hexavalent) in RI	$ 0.30	$ 0.30	$ 0.78	$ 2.84
‐ IPV vaccine patch in RI or SIAs	$ 0.95	$ 0.95	$ 2.51	$ 3.18
‐ IPV intradermal device (incremental)	$ 0.30	$ 0.30	$ 0.30	NA
Effective vaccine wastage				
‐ OPV in RI	20%	20%	15%	10%*
‐ IPV in RI	15%	15%	10%	5%
‐ IPV in IPV/OPV or OPV+IPV RI	20%	20%	15%	10%
‐ IPV, fractional, in RI	30%	30%	20%	NA
‐ IPV, fractional, in IPV/OPV or OPV+IPV RI	40%	40%	20%	NA
‐ OPV or IPV in SIAs	15%	15%	10%	10%*
‐ IPV, vaccine patch	2%	2%	1%	1%
Treatment costs per case	$ 711	$ 7,110	$ 71,100	$ 711,000
Life expectancy at birth (years, population weighted)	64.1	68.8	75.9	81.0
Disability‐adjusted life‐years (DALY) per case	13.2	13.5	13.9	14.1
$ per DALY based on gross national income (GNI) per capita (Atlas method, 2018 current estimate adjusted to US$2019, WBIL population weighted)	$ 866	$ 2,310	$ 9,140	$ 45,600
Societal willingness‐to‐pay per case avoided (based on GNI)	$ 11,300	$ 30,800	$ 125,000	$ 634,000

DALY = disability‐adjusted life year; GNI = gross national income; HI = high income; IPV = inactivated poliovirus vaccine; L =, low income; LMI = lower middle income; N/A = not applicable; OPV = oral poliovirus vaccine; oSIA = outbreak response SIA; pSIA = planned, preventive SIA; RI = routine immunization; SIA = supplemental immunization activity; UMI = upper middle income; WBIL = World Bank Income Level.

**Note**: ^*^Estimate for HI included for theoretical comparison (see text).

Table II shows our assumption of continued support of tiered pricing for polio vaccines, which play a key role in poliovirus vaccine demand and availability, particularly for IPV. In addition, on top of relatively lower income countries accessing IPV at lower prices, some countries also receive external financial support to pay for IPV purchase and administration, which changes IPV from an otherwise economically unattractive vaccine into a subsidized option for which cost matters less. However, countries receiving external financial support for IPV should not expect this to continue in perpetuity. Those countries that want to use IPV long term will need to include it in their national budgets at some point. In addition, many countries currently use both OPV and IPV in anticipation of complete OPV cessation. However, if OPV use continues in the long term (perhaps in perpetuity for serotype 1) (Kalkowska et al., 2020a), then this will most likely lead to a significant decrease in demand for the much more expensive and much more difficult to administer IPV.

We continue to vary vaccine administration costs for the different vaccine formulations, which differs from some recent cost analyses (Zimmermann et al., 2020). We previously assumed higher (Duintjer Tebbens, Pallansch, Wassalik, et al., 2015) or higher and the same (Duintjer Tebbens & Thompson, 2016) administration costs for outbreak response SIAs (oSIAs) than for preventive, planned SIAs (pSIAs), as shown in Table I. However, tracking of costs for different types of SIAs remains limited, and we now assume the same costs per administered OPV SIA dose in Table II for oSIAs and pSIAs. We continue to assume sharing of the administration costs over all of the antigens in the IPV‐containing combination vaccine, which we assume includes six components (i.e., hexavalent). In Table II, we also note that the use of a hexavalent IPV‐containing vaccine will likely require more doses of IPV in the RI schedule for some countries.

In our prior analyses (Duintjer Tebbens, Pallansch, Wassalik, et al., 2015; Thompson et al., 2008), we used a 3% discount rate for future economic and health costs to report net present values, based on available guidance (World Health Organization, 2008). A recent review highlights some of the controversies and implications of different assumptions for discount rates (Attema, Brouwer, & Claxton, 2018). Recent WHO guidance suggests reporting results using (i) discounting at a 3% rate for economic costs (but not health costs) and also (ii) at a rate of 3% both health and economic costs (World Health Organization, 2019b). We also included estimated values for productivity gains associated with disability‐adjusted live years (DALYs) saved by assuming a monetary value of the population‐weighted WBIL per capita gross national income (GNI) per DALY lost (Duintjer Tebbens et al., 2011; Duintjer Tebbens, Pallansch, Wassalik, et al., 2015). Recent health economic modeling suggests that assuming the per capita gross domestic product (GDP) per DALY may overestimate actual national willingness to pay for health interventions and encourages the use of country‐specific estimates (Ochalek, Lomas, & Claxton, 2018). Current WHO guidance allows for the use of GNI per capita to value avoided productivity losses (e.g., willingness‐to‐pay for DALYs saved) (World Health Organization, 2019b), so we continue to use this approach. However, we note that future analyses may also want to consider estimates for WBILs that account for health opportunity costs.

Table III summarizes the costs per immunization contact by type that result from application of the inputs in Table II. We refer to these cost inputs as per contact because some RI schedules include OPV+IPV, which deliver both and an OPV and an IPV dose at a single contact. Table III shows the vaccine costs with adjustment for wastage, the administration costs, and the total costs by WBIL.

**Table III:** Summary of Costs in 2019 US Dollars (US$2019) by Type of Immunization Contact by World Bank Income Level

Cost inputs by WBIL (US$2019)	Vaccine costs per contact (w/wastage)				Administration cost per contact				Total cost per contact			
Contact type	LI	LMI	UMI	HI	LI	LMI	UMI	HI	LI	LMI	UMI	HI
OPV RI	0.19	0.19	0.39	9.72*	0.95	0.95	2.51	3.18*	1.14	1.14	2.90	12.90*
OPV+IPV (3rd RI dose), full	3.31	3.50	5.98	NA	1.95	1.95	5.51	NA	5.26	5.45	11.49	NA
OPV+IPV (3rd RI dose), fractional with needle	1.02	1.07	1.57	NA	1.95	1.95	5.51	NA	2.97	3.02	7.09	NA
OPV+IPV (3rd RI dose), fractional with device	1.02	1.07	1.57	NA	2.25	2.25	5.81	NA	3.27	3.32	7.39	NA
OPV+IPV (3rd dose), vaccine patch	1.92	1.95	3.37	NA	1.90	1.90	5.02	NA	3.82	3.85	8.39	NA
nOPV RI	0.38	0.38	0.77	9.72*	0.95	0.95	2.51	3.18*	1.32	1.32	3.29	12.90*
nOPV+IPV (3rd RI dose), full	3.50	3.69	6.36	NA	1.95	1.95	5.51	NA	5.45	5.64	11.87	NA
nOPV+IPV (3rd RI dose), fractional with needle	1.21	1.26	1.96	NA	1.95	1.95	5.51	NA	3.16	3.21	7.47	NA
nOPV+IPV (3rd RI dose), fractional with device	1.21	1.26	1.96	NA	2.25	2.25	5.81	NA	3.46	3.51	7.77	NA
nOPV+IPV (3rd dose), vaccine patch	2.11	2.14	3.75	NA	1.90	1.90	5.02	NA	4.00	4.04	8.78	NA
IPV RI, single full	2.94	3.12	5.28	15.02	1.78	1.78	4.69	17.06	4.72	4.87	9.97	32.08
IPV RI, single fractional with needle	0.71	0.76	1.19	NA	1.78	1.78	4.69	NA	2.49	2.53	5.88	NA
IPV RI, single fractional with device	0.71	0.76	1.19	NA	2.08	2.08	4.99	NA	2.79	2.83	6.18	NA
IPV RI, combo dose	4.38	5.00	7.75	30.12	0.30	0.30	0.78	2.84	4.67	5.30	8.53	32.96
IPV, vaccine patch, RI or SIA	1.73	1.77	2.98	27.38	0.95	0.95	2.51	3.18	2.68	2.71	5.49	30.56
OPV in pSIA	0.18	0.18	0.37	9.72*	0.95	0.95	2.51	3.18*	1.12	1.12	2.88	12.90*
OPV in oSIA	0.18	0.18	0.37	9.72*	0.95	0.95	2.51	3.18*	1.12	1.12	2.88	12.90*
nOPV in pSIA	0.35	0.35	0.73	9.72*	0.95	0.95	2.51	3.18*	1.30	1.30	3.24	12.90*
nOPV in oSIA	0.35	0.35	0.73	9.72*	0.95	0.95	2.51	3.18*	1.30	1.30	3.24	12.90*
IPV SIA, single full	2.94	3.12	5.28	15.86	1.78	1.78	4.69	17.06	4.72	4.89	9.97	32.91
IPV SIA, single fractional with needle	0.59	0.62	1.06	NA	1.78	1.78	4.69	NA	2.36	2.40	5.75	NA
IPV SIA, single fractional with device	0.59	0.62	1.06	NA	2.08	2.08	4.69	NA	2.66	2.70	6.05	NA

HI = high income; IPV = inactivated poliovirus vaccine; LI = low income; LMI = lower middle income; NA = not applicable; nOPV = novel oral poliovirus vaccine; OPV = oral poliovirus vaccine; RI = routine immunization; SIA = supplemental immunization activity; UMI = upper middle‐income; WBIL = World Bank Income Level.

**Note**: ^*^Estimate for HI included for theoretical comparison (see text).

Table IV summarizes the resulting cost per fully immunized child by WBIL by RI schedule or SIA vaccine formulation. The estimates in Table IV include the use of nOPV in RI or in SIAs, although the timing of the availability, actual costs, and properties of any nOPV remain uncertain. Similarly, Table IV includes the option of IPV delivered using a vaccine patch, although no such product currently exists. Table IV provides these estimates to support prospective modeling that may help policy makers evaluate the benefits of investments in the development of new vaccine formulations.

**Table IV:** Estimated Costs in 2019 US Dollars (US$2019) Per Fully Immunized Child by Routine Immunization (RI) Schedule or Supplemental Immunization Activity (SIA) and Vaccine Formulation by WBIL

	Number of Contacts			Cost (US$2019) by WBIL			
RI schedule	OPV or nOPV	OPV + IPV	IPV	LI	LMI	UMI	HI
OPV only	3	0	0	3.41	3.41	8.69	38.71*
OPV+IPV, full	2	1	0	7.53	7.72	17.28	NA
OPV+IPV + 2nd IPV dose, full	2	1	1	12.25	12.61	27.25	NA
OPV+IPV, fractional	2	1	0	5.24	5.29	12.88	NA
OPV+IPV + 2nd IPV dose, fractional	2	1	1	7.73	7.82	18.76	NA
OPV+IPV, fractional device	2	1	0	5.54	5.59	13.18	NA
OPV+IPV + 2nd IPV dose, fractional device	2	1	1	8.33	8.42	19.36	NA
OPV+IPV, patch	2	1	0	6.09	6.12	14.19	NA
OPV+IPV + 2nd IPV dose, patch	2	1	1	8.77	8.83	19.68	NA
IPV/OPV	2	0	2	11.71	12.06	25.73	89.96
IPV/OPV	1	0	3	15.29	15.82	32.80	109.13
nOPV only	3	0	0	3.97	3.97	9.86	38.71*
nOPV+IPV, full	2	1	0	8.09	8.28	18.45	NA
nOPV+IPV + 2nd IPV dose, full	2	1	1	12.81	13.17	28.41	NA
nOPV+IPV, fractional	2	1	0	5.80	5.85	14.04	NA
nOPV+IPV + 2nd IPV dose, fractional	2	1	1	8.29	8.38	19.92	NA
nOPV+IPV, fractional device	2	1	0	6.10	6.15	14.34	NA
nOPV+IPV + 2nd IPV dose, fractional device	2	1	1	8.89	8.98	20.52	NA
nOPV+IPV, patch	2	1	0	6.65	6.68	15.35	NA
nOPV+IPV + 2nd IPV dose, patch	2	1	1	9.33	9.39	20.84	NA
IPV/nOPV	2	0	2	12.08	12.43	26.51	89.96
IPV/nOPV	1	0	3	15.48	16.01	33.19	109.13
IPV‐only, standalone	0	0	2	9.44	9.79	19.49	64.15
IPV‐only, standalone	0	0	3	14.15	14.68	29.90	96.23
IPV‐only, fractional	0	0	2	4.98	5.07	11.76	NA
IPV‐only, fractional	0	0	3	7.47	7.60	17.63	NA
IPV‐only, fractional, device	0	0	2	6.54	6.64	14.77	NA
IPV‐only, fractional, device	0	0	3	9.81	9.96	22.16	NA
IPV‐only, patch	0	0	2	5.36	5.43	10.98	61.13
IPV‐only, patch	0	0	3	8.05	8.14	16.47	91.69
IPV‐only, combination	0	0	4	18.68	21.18	34.11	131.85
**SIA dose**							
OPV in pSIA	1	0	0	1.14	1.14	2.90	12.90*
OPV in oSIA	1	0	0	1.14	1.14	2.90	12.90*
nOPV in pSIA	1	0	0	1.32	1.32	3.29	12.90*
nOPV in oSIA	1	0	0	1.32	1.32	3.29	12.90*
IPV SIA, single full	0	0	1	4.72	4.89	9.24	26.39
IPV SIA, single fractional with needle	0	0	1	2.36	2.40	5.75	NA
IPV SIA, single fractional with device	0	0	1	2.66	2.70	6.05	NA
IPV SIA, vaccine patch	0	0	1	2.68	2.71	5.49	30.56

HI = high income; IPV = inactivated poliovirus vaccine; LI = low income; LMI = lower middle income; NA = not applicable; nOPV = novel oral poliovirus vaccine; OPV = oral poliovirus vaccine; RI = routine immunization; SIA = supplemental immunization activity; UMI = upper middle income; WBIL = World Bank Income Level.

**Note**: ^*^Estimate for HI included for theoretical comparison (see text).

## 6 DISCUSSION

Currently and recently endemic countries require ongoing use of OPV to stop and prevent WPV and/or cVDPV transmission, and the benefits of IPV use in these countries currently appear small relative to the benefits of the OPV use (i.e., IPV use contributes relatively little to overall population immunity to transmission and comes at a relatively higher financial cost). In contrast, in relatively higher income countries with strong health systems that achieve high coverage and eliminated indigenous WPV transmission many years ago, IPV plays a more significant role by preventing VAPP and protection from any live poliovirus importations. After successful WPV eradication and cessation of OPV use, IPV would represent the only polio vaccine available to protect otherwise unprotected individuals from paralysis. However, if OPV cessation cannot occur successfully, and any OPV use must continue, then OPV would most‐likely represent a more cost‐effective option than IPV for current OPV‐using countries. Cases of paralysis prevented by IPV use will save incremental costs on treatment and prevent incremental productivity losses, but only for fully susceptible individuals who receive only the IPV. The amount of prevention depends on the risks (i.e., exposure to virulent live polioviruses, which during the polio endgame should be theoretically zero and practically very low), IPV coverage (i.e., lower coverage implies lower benefits of the IPV insurance), and the timing of IPV doses in the schedule (i.e., VAPP prevention for schedules that deliver IPV before OPV).

The impacts of IPV use on outbreak response remain uncertain. The prevention of paralytic cases by IPV may delay the detection of paralytic cases associated with viral transmission and allow the virus to spread further prior to detection and response (necessitating a larger response), while individuals with IPV‐only protection who receive OPV in an outbreak response may participate somewhat less in transmission than if they were completely unvaccinated. Again, the benefits of IPV will depend on coverage. If live poliovirus transmission starts in a country with high IPV coverage (e.g., Israel), then the IPV may prevent all or most cases of paralysis. However, in countries that use IPV in RI that achieve low coverage, the IPV use will not prevent most cases. In most countries, IPV use will not likely impact the need to use OPV for outbreak response. The likelihood of future outbreaks, and thus the potential benefits of IPV, depend on the quality of risk management activities undertaken by the GPEI now and in the future. For example, if the GPEI partners and countries effectively prevent cVDPVs that might arise after OPV cessation, then the benefits of IPV preventing cases associated with cVDPVs will approach zero. In contrast, if the GPEI and countries do not manage risks prior to OPV cessation well, then the IPV used will prevent more cases and associated costs, albeit without reducing the likely needs for OPV for outbreak response or the need to restart OPV.

Gavi became a significant stakeholder in the polio endgame with its support of IPV introduction in OPV‐using Gavi‐eligible countries starting in 2015. The initial intent of the support focused on accelerating adoption of IPV and then transitioning the costs of IPV to the countries over time. However, more recently, Gavi has significantly expanded its mission and shifted its focus from vaccine introduction to equity. Gavi IPV funding for 2021–2025 signals a commitment to global equity in vaccine delivery for IPV, independent of any risks or benefits. So long as children in relatively higher income countries receive IPV, arguments can be made about the need to provide equitable access to IPV for children in relatively lower income countries. Gavi IPV funding would help to ensure access to affordable IPV for its supported countries, although it will not necessarily ensure access to affordable IPV for children in non‐Gavi‐eligible countries of relatively lower income. With respect to equity, the fundamental issue is whether a commitment to supporting IPV by Gavi implies that Gavi's donors will assume the burden of paying for IPV and thus guaranteeing that children in Gavi‐eligible countries receive IPV, or if it implies access to affordable IPV (i.e., ensuring access to a low‐cost vaccine) without guaranteeing purchase the vaccine (i.e., expecting countries that want IPV to take advantage of the low cost, but to raise their own financing). The recent Gavi decision to expand its support of IPV will likely create expectations for Gavi donors to provide support for IPV for Gavi‐supported countries so long as IPV remains recommended (i.e., through the 2021–2025 and 2026–2030 funding periods and potentially beyond based on the WHO SAGE recommendation of at least two doses of IPV for at least 10 years after global OPV withdrawal). Notably, this may imply support for and use of a vaccine that the countries would not otherwise use. In addition, national financial support for IPV in OPV‐using countries would most likely depend on the timing of complete cessation of OPV, since countries will need to pay for OPV in RI (and in SIAs in some countries) until that time.

The expected use of IPV by relatively higher income countries long term to prevent adverse events associated with iVDPVs and/or containment breaches reflects: (1) their relatively higher risks of these events, (2) their expectation of continuing to maintain live poliovirus stocks (e.g., for IPV production and/or research), (3) the increased integration of IPV into combination vaccines, (4) the relatively higher treatment costs associated with polio cases, including VAPP, and (5) their greater willingness‐to‐pay to avoid polio cases compared to relatively lower income countries. In addition, longer use of IPV by relatively lower income countries will likely increase the risks of reintroduction associated with containment breaches (i.e., pressure on price to support relatively lower income countries leads to greater pressure to produce IPV in or near these countries, which increases the risks associated with IPV production) (Duintjer Tebbens, Pallansch, Wassalik, et al., 2015).

Although eradication represents the ultimate opportunity to provide global equity by permanently stopping and preventing exposure to poliovirus threats for everyone and everywhere, in the name of equity and without consideration of the economics or opportunity costs, significant resources have and could be spent for long term very high global control with two doses of IPV, in addition to the OPV needed. The nonrival and nonexclusive benefits associated with eradication make it a global public good, and the benefits of the good depend on its preservation, but IPV plays little expected role in preserving WPV eradication in Gavi‐eligible countries, once achieved. Notably, IPV does not fit into the typical Gavi evaluation criteria (i.e., lives saved, value for money) or return on investment criteria typically considered by finance ministers. Instead, IPV use after WPV eradication represents a form of insurance, which offers to protect otherwise‐susceptible children who receive it from potential paralysis if they subsequently get exposed to live polioviruses and to save the associated treatment costs and productivity losses. Eradication should minimize the risks of infection, and in theory, the risks should approach zero, but in practice, the risks will remain nonzero so long as live polioviruses exist.

As with all forecasts, these estimates come with limitations. First, although we do not anticipate the costs of poliovirus vaccines going down (i.e., production at scale already exists, containment costs continue to increase), but some new innovation in vaccine production technology could lower costs in ways that we do not foresee. Second, although we do not anticipate the costs increasing much beyond our estimates, the costs could increase due to other factors, including significant disruption of the vaccine market by COVID‐19. In addition, the current development of nOPV may lead to a substantial shift in the polio endgame that does not include OPV cessation or require IPV use in all countries.

Twenty years after the initial target for achieving polio eradication, the costs of the vaccines and expectations for their prolonged use continue to increase. The increases in vaccine costs and delay in achieving eradication will impact the overall economics of the GPEI, and further studies should reevaluate using these updated cost estimates and updated poliovirus transmission and OPV evolution modeling (Kalkowska et al., 2020a). The addition of IPV to all national immunization schedules substantially increased the costs of the polio endgame, and will likely impact national immunization budgets in most countries for much longer than many national health leaders may expect. The increased costs of poliovirus vaccines increase the financial risks faced by the GPEI.

## FUNDING

The manuscript was funded by a grant from the Bill and Melinda Gates Foundation [OPP1129391/INV‐009333].
